# A novel SNP-based multiplex PCR profiling divulges contemporary genetic patterns in Haitian variant *Vibrio cholerae* O1 clinical isolates

**DOI:** 10.1128/spectrum.02661-25

**Published:** 2026-09-21

**Authors:** Prosenjit Samanta, Priyanka Ghosh, Goutam Chowdhury, Sreeja Shaw, Arindam Naha, Deboleena Roy, Ayumu Ohno, Shin-ichi Miyoshi, Thandavarayan Ramamurthy, Asish Kumar Mukhopadhyay

**Affiliations:** 1 Division of Bacteriology, ICMR-National Institute for Research in Bacterial Infections 30170 , Kolkata, India; 2 Collaborative Research Centre of Okayama University for Infectious Diseases, ICMR-National Institute for Research in Bacterial Infections 30170 , Kolkata, India; 3 Graduate School of Medicine, Dentistry and Pharmaceutical Sciences, Okayama University 12997, Okayama, Japan; Weill Cornell Medicine, New York, New York, USA

**Keywords:** cholera, *Vibrio cholerae*, multiplex PCR, *ctxB*, *tcpA*, *rtxA*

## Abstract

The massive cholera outbreak in Haiti in 2010 has drawn the attention of both medical professionals and public health officials worldwide. The hypervirulent *Vibrio cholerae* O1 El Tor strains responsible for this outbreak were not only confined to Haiti but also spread quickly to other cholera-prone regions. Therefore, tracking and understanding these strains in endemic areas have become crucial for timely and effective treatment strategies. The presence of specific genetic mutations in key virulence-related genes*—ctxB* (cholera enterotoxin subunit B), *tcpA* (toxin-coregulated pilin), and *rtxA* (repeats-in-toxin)—makes these Haitian strains distinctive. These genes contain single nucleotide polymorphisms, which act as unique markers for identifying the Haitian variant. However, current detection methods using allele-specific simplex PCRs are time-consuming and can delay critical outbreak responses. To address this, we developed new Haitian allele-specific primers targeting *rtxA* and *tcpA* and combined them with an existing *ctxB*-specific PCR into a single multiplex PCR assay. We tested this new multiplex PCR on 250 clinical *V. cholerae* O1 isolates collected from diarrheal patients in Kolkata, India, between 2002 and 2019. Interestingly, we noticed that the first appearance of Haitian *tcpA, rtxA,* and *ctxB* alleles was in 2003, 2004, and 2006, respectively, years before the Haitian outbreak occurred. Our results matched perfectly with the conventional simplex PCRs and demonstrated 100% sensitivity and specificity. Given the global spread of these Haitian-like *V. cholerae* strains, this multiplex PCR provides a fast, reliable tool for identifying cholera infection and gaining valuable epidemiological insights.

**IMPORTANCE:**

Timely identification of pathogens causing life-threatening diseases is vital in clinical and public health contexts. The emergence and global spread of Haitian *Vibrio cholerae* O1 El Tor strains during cholera outbreaks highlight the urgent need for rapid and reliable diagnostics. To address this, we developed a novel, highly sensitive multiplex PCR assay that delivers results within 2 h from identified isolates.This assay enables efficient detection and differentiation of Haitian *V. cholerae* strains. Extensive evaluation with a large panel of isolates confirmed its effectiveness in rapid identification and provided valuable insights into the temporal evolution of key virulence-associated genes. Specifically, the assay targets key genetic markers *ctxB*, *tcpA*, and *rtxA*, which are critical for monitoring shifts in virulence and transmission potential. Our findings establish this multiplex PCR as a robust tool for surveillance and diagnosis, especially valuable in outbreak settings where timely intervention is paramount.

## INTRODUCTION

Cholera has predominantly affected developing countries in Asia and Africa for decades, yet it continues to pose significant public health challenges globally (1–6). In 2024 alone, over 30 countries reported cholera outbreaks, prompting the World Health Organization to declare the highest level of emergency (7). The disease is caused by *Vibrio cholerae*, a pathogen renowned for its environmental adaptability, which is largely driven by frequent genetic rearrangements (8). These genetic shifts have led to the emergence of hypervirulent variants, responsible for widespread and severe outbreaks (9, 10). *V. cholerae* has been implicated in seven pandemics, with the ongoing seventh pandemic beginning in 1961 (4, 11–13). The earlier six pandemics were largely caused by the classical biotype, known for its higher virulence but modest environmental persistence (11, 14). In contrast, the ongoing pandemic is attributed to the El Tor biotype, which has demonstrated enhanced global transmission through three distinct waves (15). The current third wave notably struck Haiti in 2010, marking the first major outbreak in the Caribbean nation in nearly a century. The scale and severity of this outbreak raised serious concerns among public health authorities (6, 15).

The 2010 Haitian outbreak is considered one of the largest in recent cholera history, resulting in over 8,000 deaths within a year (6). The outbreak introduced a variant of the *V. cholerae* O1 El Tor strain with notable chromosomal alterations (16). Whole-genome sequencing revealed multiple single nucleotide polymorphisms (SNPs), particularly in key virulence genes, including *ctxB*, *tcpA*, and *rtxA*—resulting in the Haitian-specific alleles *ctxB7*, *tcpA^CIRS^*, and *rtxA^o^*, respectively (17–19). The *tcpA^CIRS^* allele contains an A266G mutation believed to enhance virulence (20–22). TcpA is crucial for intestinal colonization and also acts as the receptor for the CTX prophage, which carries the cholera toxin genes (23–27).

Colonization of *V. cholerae* in the host intestine facilitates the secretion of cholera toxin, one of the major virulence factors responsible for disease pathogenesis. This toxin is encoded by the CTX prophage and comprises an enzymatically active A subunit and a polymorphic B subunit (CTB), the latter of which exists in at least 13 distinct genotypes (28, 29). Among these, the classical CTB type encoded by *ctxB1* has been reported to exhibit greater toxicity than other variants (30). A recently emerged variant, *ctxB7* (also known as the Haitian *ctxB*), closely resembles *ctxB1* but harbors a single nucleotide substitution at position 58 (G58T) within the signal peptide region. This mutation has been associated with a hypervirulent phenotype in animal models (17, 31). In addition to cholera toxin, the *rtxA* encodes a large multifunctional cytotoxin with several key domains, including an actin cross-linking domain, a Rho GTPase inactivation domain, and an alpha/beta-hydrolase domain, which together damage the host cytoskeleton and cellular architecture (32, 33). Notably, a point mutation (G13602A) within the *rtxA* has been identified in the El Tor biotype, resulting in a premature stop codon and generating the Haitian *rtxA* variant (32). This mutation prevents the actin cross-linking function, rendering the cytotoxin partially inactive. Despite its reduced cytotoxic activity, the Haitian *rtxA* variant is hypothesized to provide a genomic background favorable for the emergence and establishment of the Haitian *ctxB* genotype (18).

The genes *ctxB*, *tcpA*, and *rtxA* are considered key determinants of *V. cholerae* pathogenesis. Although the genomic alterations in these loci gained attention following the Haitian cholera outbreak, evidence suggests that these variants existed well before the epidemic (18). Phylogenetic analyses have revealed that Haitian *V. cholerae* strains share a common lineage with the South Asian Wave 3 strains (34). Notably, the Haitian variant *tcpA^CIRS^* and *ctxB7* alleles were also reported in *V. cholerae* identified from cholera outbreaks in Nigeria (2, 35), southwestern India in 2012 (36), and Mexico in 2013 (36). Our previous studies have documented the presence of *tcpA^CIRS^*, *ctxB7,* and *rtxA^o^* alleles in *V. cholerae* isolates from Kolkata and other regions of India (8, 17–19, 37). Therefore, the rapid spread of Haitian variant in Asia, Africa, and the Americas (5, 38, 39) over decades has motivated us to develop a quick characterization tool for tracking the emergence and dissemination of *V. cholerae* with Haitian genetic attributes.

In this study, we present a multiplex PCR assay designed for the efficient identification of *V. cholerae* isolates carrying the Haitian-specific *tcpA^CIRS^*, *ctxB7*, and *rtxA^o^* alleles with high sensitivity and specificity. Our findings highlight the utility of this multiplex PCR assay as a reliable and effective tool for the surveillance and detection of hypervirulent *V. cholerae* isolates, particularly in outbreak situations where rapid intervention is critical.

## MATERIALS AND METHODS

### Bacterial cultures and growth conditions

Representative clinical *V. cholerae* isolated during 2002–2019 from cholera patients admitted to the Infectious Diseases and Beliaghata General Hospital (IDH), Kolkata, India, were included in this study. Standard reference strains O395, N16961, and El-1786 were used as controls for classical, El Tor, and Haitian variants, respectively. The test isolates were grown at 37°C on thiosulfate-citrate-bile salts-sucrose medium (Oxoid, UK) for the selection of typical *V. cholerae* and subsequently sub-cultured on Luria-Bertani (LB) agar plates (Difco, BD, USA) for downstream experiments. Serogroup determination was performed using slide agglutination test with O1/O139-specific polyvalent antiserum (Denka Seiken, Japan).

### DNA extraction and dilution

Genomic DNA was extracted from *V. cholerae* isolates grown on LB agar using a standard phenol-chloroform method, as described previously (40). Briefly, one loop full of bacterial cultures was suspended in 200 µL of Tris-EDTA buffer and mixed with an equal volume of phenol-chloroform-isoamyl alcohol (25:24:1), followed by centrifugation at 13,000 rpm for 15 min. The upper aqueous layer was separated and mixed with an equal volume of chloroform-isoamyl alcohol (24:1) and then centrifuged again at the previously mentioned speed. The upper aqueous layer was collected and diluted to a desired concentration for the downstream PCR assay. Alternatively, for rapid screening, a single colony was suspended in 20 µL of sterile water, and 4 µL of the suspension was directly added to the PCR reaction mix as a template. During outbreak situations requiring rapid diagnostics, DNA from stool samples can be directly isolated using commercially available stool DNA extraction kits (e.g., Qiagen and Omega), with total processing time as short as 30 min, enabling prompt multiplex PCR detection.

### Multiplex PCR assay

**Fig 1:**
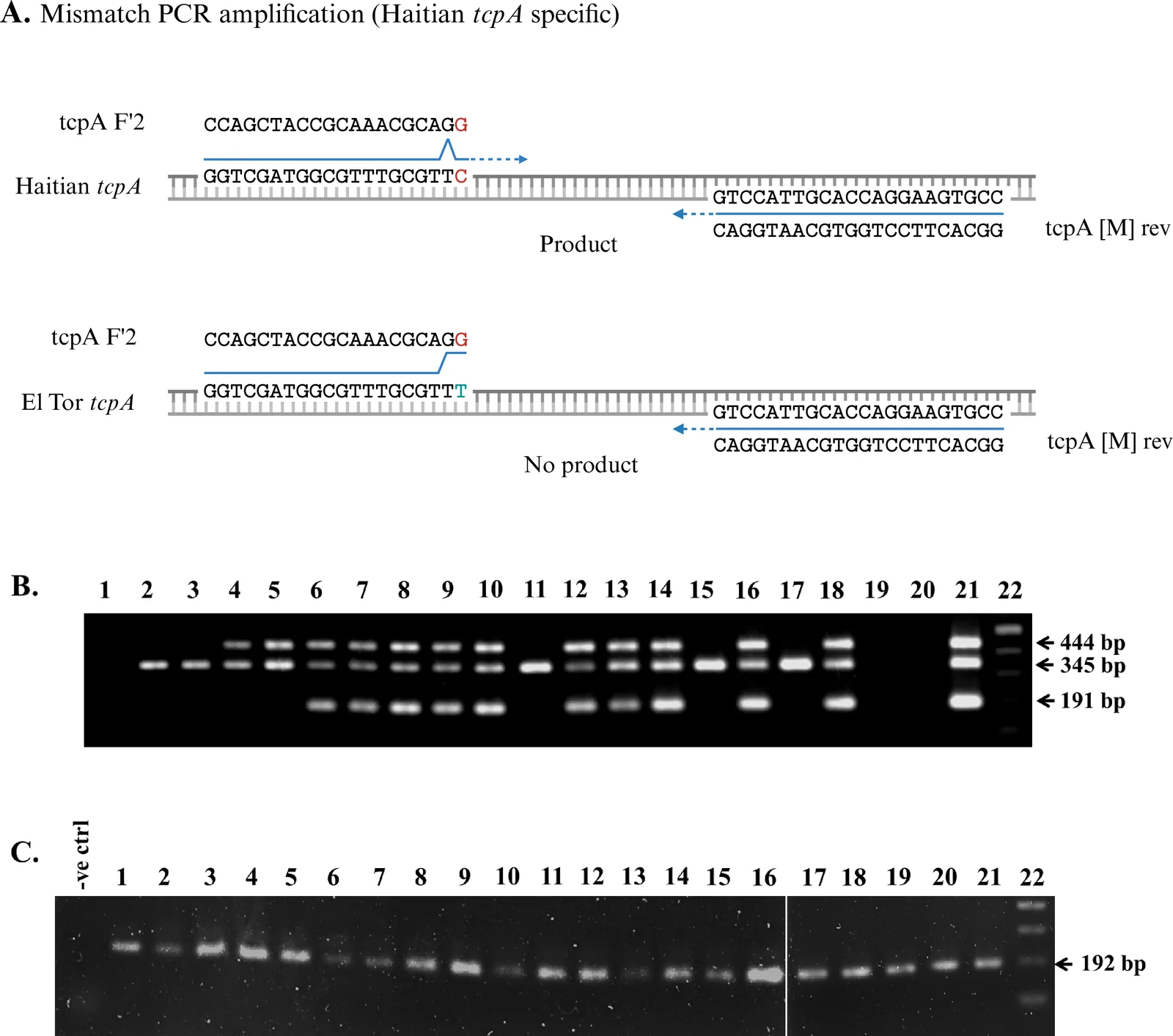
Multiplex PCR assay for the detection of Haitian variant *rtxA*, *tcpA,* and *ctxB* alleles in *V. cholerae* O1 isolates in Kolkata. (**A**) Mismatch PCR amplification approach used to detect known single nucleotide mismatches in Haitian variant alleles of *ctxB*, *tcpA,* and *rtxA* using primer specificity. Here, for example, the tcpAF′2 primer is shown to target the Haitian variant *tcpA* allele and produce an amplicon while failing to amplify the El Tor *tcpA* allele due to significant nucleotide mismatches (indicated in red and green), using a common reverse primer, tcpA[M]rev. A part of the figure was created with BioRender.com. (**B**) Representative agarose gel image of multiplex PCR to detect the Haitian variant *rtxA* allele, *tcpA* allele, and *ctxB* allele in representative *Vibrio cholerae* O1 strains of Kolkata using primer sets rtxA(mlt)F/rtxA(mlt)R (amplicon size, 444 bp) for Haitian variant *rtxA*, tcpAF′2/tcpA[M]rev (amplicon size, 345 bp) for Haitian variant *tcpA,* and ctxB-F3/ctxB Rv-Cla (amplicon size, 191 bp) for Haitian variant *ctxB* allele indicated the gradual appearance of Haitian variant alleles through years. (**C**) *Vibrio cholerae* O1-specific PCR targeting the *rfb*O1 locus to serve as a loading control for all the strains tested. For both the panels, lanes 1–18 represent BD155 (2002), SC46 (2003), SC68 (2003), J3752 (2004), K12398 (2005), L16899 (2006), IDH00745 (2008), IDH03044 (2010), IDH04415 (2012), IDH07230 (2014), IDH08338 (2015), IDH08413 (2015), IDH08890 (2016), IDH10445 (2017), IDH10469 (2017), IDH11596 (2018), IDH11867 (2018), and IDH12457 (2019), respectively; lane 19, O395; lane 20, N16961; lane 21, EL-1786; and lane 22, 100-bp size ladder (New England Biolabs Inc., Beverly, MA, USA). Additionally, panel C contains *Escherichia coli* K-12 genomic DNA as a negative control (−ve ctrl) for the *rfb*O1 PCR.

A multiplex PCR assay was developed to simultaneously detect three target genes with their respective genotypes. For Haitian *tcpA* amplification, the *tcpA^CIRS^*-specific forward primer (tcpA-F′2) and a newly designed reverse primer (tcpA[M]rev) were employed (Fig. 1A). Detection of Haitian *ctxB7* was carried out using a pre-existing forward primer (ctxB-F3) in combination with the reverse primer (Rv-cla). Additionally, a novel primer pair [rtxA(mlt)F and rtxA(mlt)R2] was designed for the amplification of the Haitian variant *rtxA* (Table 1). The new primers were optimized to produce distinct, easily distinguishable amplicons on agarose gel electrophoresis, and their melting temperatures (*T*_*m*_) were standardized to 58°C to align with the *T*_*m*_ of the pre-existing primers used in the assay.

**TABLE 1:** Primer sequences and annealing temperatures used for PCR in this study

Primer	Sequences (5′ – 3′)	Annealing temperature (°C)	References
ctxB-F3	GTTTTACTATCTTCAGCATATGCGA	58	(17)
Rv-cla	CCTGGTACTTCTACTTGAAACG	60	(17)
tcpA-F′2	CCAGCTACCGCAAACGCAGG	56	(19)
tcpA[M]rev	GGCACTTCCTGGTGCAATGGAC	58	This study
rtxA(mlt)F	GCACGGATGTGGACAGCTTTG	58	This study
rtxA(mlt)R2	TCCTTTTTTGTGAACCACGTCTGCT	58	This study
tcpA EL-Rev	CCGACTGTAATTGCGAATGC	56	(19)
rtxA-F	ATCGGAATGAGTGAGAAAGACC	54	(18)
rtxA-R2	TGTGAACCACGTCTGCT	54	(18)
ctxB-F4	GTTTTACTATCTTCAGCATATGCGC	60	(17)
tcpA-F1	CCAGCTACCGCAAACGCAGA	56	(19)
rtxAR1	TGTGAACCACGTCTGCC	54	(18)
rfb O1 cluster-F	GTTTCACTGAACAGATGGG	54	(41)
rfb O1 cluster-R	GGTCATCTGTAAGTACAAC	54	(41)

The PCR reactions were performed in a 20 µL volume containing 2 µL of 10× PCR buffer with Mg²^+^ (TaKaRa Bio, Japan), 4 µL (~50 ng) of template DNA, 0.2 µL (100 nM) of each forward and reverse primers for the *rtxA* and *tcpA* genes, 0.6 µL (300 nM) of each forward and reverse primers for the *ctxB* gene, 1.6 µL of dNTP mix (200 µM each; TaKaRa Bio), and 0.2 µL (1 unit) of Taq DNA polymerase (TaKaRa Bio).

The cycling conditions were optimized to ensure both high specificity and sensitivity: initial denaturation at 96°C for 2 min, followed by 28 cycles of denaturation at 94°C for 30 s, annealing at 58°C for 25 s, and extension at 72°C for 25 s, with a final extension at 72°C for 1 min. This standardized multiplex PCR successfully differentiated the three allelic subtypes within a single reaction mixture.

### Simplex PCR assay

To validate the specificity of the multiplex PCR, simplex PCR assays targeting three Haitian allele-specific genes (*tcpA*, *ctxB*, and *rtxA*) were performed. The amplification of Haitian variant genotypes of *ctxB*, *tcpA*, and *rtxA* was performed using primer pairs ctxB-F3/Rv-cla, tcpA-F′2/tcpA El-Rev, and rtxA-F/rtxA-R2, respectively, following previously published protocols (37). In addition to the Haitian alleles, the other genotypes that are co-circulating with the Haitian strains were also monitored for each of the three genes. For the classical *ctxB* allele, ctxB-F4/Rv-cla was used. Similarly, amplification of the El Tor variants of *tcpA* and *rtxA* was performed using primer pairs tcpA-F1/tcpA El-Rev and rtxA-F/rtxA-R1, respectively (Table 1) (37).

### Statistical analysis

A conventional 2 × 2 contingency table was employed to evaluate the sensitivity and specificity of the multiplex PCR assay in comparison with the previously established simplex PCR assay for the detection of Haitian genotypes. True positives (TPs) were defined as samples that tested positive by both multiplex and simplex PCR assays. False positives (FPs) referred to samples that were positive by multiplex PCR but negative by simplex PCR. Conversely, false negatives (FNs) are considered for results positive by simplex PCR, but negative by the multiplex PCR method. True negatives (TNs) were recorded with negative results in both the PCR methods. Sensitivity was calculated by dividing TP by the sum of TP and FN. Specificity was calculated by dividing TN by the sum of TN and FP (42). Statistical significance (*P* value) was calculated using two-tailed Fisher’s Exact test, performed in GraphPad Prism version 6.

## RESULTS

### Multiplex PCR enhances detection efficiency and specificity for Haitian variant *V. cholerae* O1

In this study, we developed a multiplex PCR assay for the rapid and specific identification of the Haitian variant strain of *V. cholerae* O1 by simultaneously targeting the *ctxB*, *tcpA*, and *rtxA*. Novel primers were designed for the *rtxA* and *tcpA* to optimize assay performance. To detect the Haitian variant of *rtxA*, a reverse primer, rtxA(mlt)R2, was designed based on a single nucleotide polymorphism site so that the reverse primer can recognize the Haitian *rtxA* sequence. The SNP base lies on the 14,602-bp region. The reverse primer is positioned in such a way that the nucleotide at the extreme 3′ end of the primer is complementary to the actual sequence found in the Haitian strains, so that it can only bind and amplify the Haitian variant *rtxA* but not the El Tor *rtxA*. Its annealing temperature was optimized to fall within 56°C–60°C, aligning with the other primers in the multiplex reaction. The corresponding forward primer, rtxA(mlt)F, was designed from a non-SNP-based common region of the *rtxA* to produce a 444-bp amplicon. For *tcpA* amplification, we employed a previously published SNP-based mismatch forward primer, tcpAF′2 (19), the 3′ end nucleotide of which is complementary to the 266^th^ nucleotide region of the Haitian *tcpA* so that it can bind and amplify only the Haitian *tcpA* but not the El Tor *tcpA* since the only difference between the two genotypes is G266A (Fig. 1A). A new reverse primer, tcpA[M]rev, was also designed encompassing a common region to pair with the tcpAF′2 and to yield a 345-bp product, with an annealing temperature of 58°C. For the detection of the Haitian *ctxB* allele, we used the established primer pair ctxB-F3/ctxB-Rv-cla (17), which produces a 191-bp product. The nucleotide specificity for the SNP lies at the 3′ end nucleotide of the forward primer ctxB-F3, which binds with the Haitian *ctxB* targeting the 58^th^ nucleotide region with high specificity but not with the classical *ctxB* allele. The multiplex PCR conditions were optimized across annealing temperatures of 56°C, 58°C, and 60°C. Clear, distinct bands were observed at 58°C, which was selected as the optimal annealing temperature. To overcome mis-annealing in the relatively low annealing temperatures at the non-specific sites, we added a deliberate mismatch just before the SNP position in the primer, creating two mismatches at the 3′ end with alleles other than the Haitian variant and thus amplification does not occur. The extension step was limited to a single cycle of 1 min to reduce the formation of nonspecific products, particularly larger amplicons exceeding 500 bp. This modification was critical to prevent the generation of a ~1 kb artifactual product that could arise due to the close genomic proximity of *ctxB* and *rtxA*, especially when using the ctxB-F3 and rtxA(mlt)R2 primers in the same reaction. The assay was validated using the Haitian variant control strain EL-1786 (*V. cholerae* O1 Ogawa, El Tor biotype), isolated from a patient in the Artibonite Department, Haiti, in October 2010. This strain yielded three distinct amplicons of 444 bp (*rtxA*), 345 bp (*tcpA*), and 191 bp (*ctxB*) (Fig. 1B). In contrast, standard *V. cholerae* O1 strains N16961 (O1 Inaba, El Tor biotype, isolated in Bangladesh, 1971) and O395 (O1 Ogawa, Classical biotype, isolated in India, 1965) produced no amplification due to substantial mismatches with the Haitian alleles and the allele-specific primers. Strains harboring all three Haitian-specific alleles showed identical triplet banding patterns to EL-1786 on 2% agarose gel. To confirm that the absence of amplification in the negative controls of the multiplex PCR was due to primer mismatches rather than insufficient genomic material, we performed a *V. cholerae* O1-specific PCR targeting the *rfb*O1 locus. All strains, including those negative in the multiplex PCR, produced the desired 192-bp amplicon (Fig. 1C). A subset of tested isolates produced one or two bands, corresponding either to *tcpA* alone or to both *rtxA* and *tcpA* (Fig. 1A), indicating partial genotypic matches to the Haitian variant.

### Multiplex PCR unveils the virulence gene profiles of circulating *V. cholerae* O1 strains in Kolkata (2002–2019)

To evaluate the reliability and consistency of the developed multiplex PCR assay, we screened a total of 250 clinical *V. cholerae* O1 isolates collected from Kolkata between 2002 and 2019. All the isolates were serologically confirmed as *V. cholerae* O1, displaying agglutination with both polyvalent O1 and monovalent Ogawa antisera. Notably, isolates from 2002 lacked all three target virulence genes associated with the Haitian genotype, indicating the absence of Haitian variant alleles during that period. A subset of s from 2003 exhibited the presence of the *tcpA* carrying the Haitian allele (*tcpA*^CIRS^), suggesting its initial emergence in the region. Between 2004 and 2005, a proportion of strains tested positive for *tcpA*^CIRS^ and the Haitian variant of *rtxA* (*rtxA^o^*), while the *ctxB* Haitian variant remained undetected. From 2006 to 2009, a limited number of isolates were found to harbor all three Haitian-type alleles—*tcpA*^CIRS^, *ctxB7*, and *rtxA^o^*—indicating the early co-circulation of these virulence determinants (Fig. 1B). A marked shift was observed from 2010 onward: nearly all the isolates collected between 2010 and 2019 contained all three Haitian variant genes, with the exception of 2015 and 2018, during which a majority of the strains exhibited this genotype.

**Fig 2:**
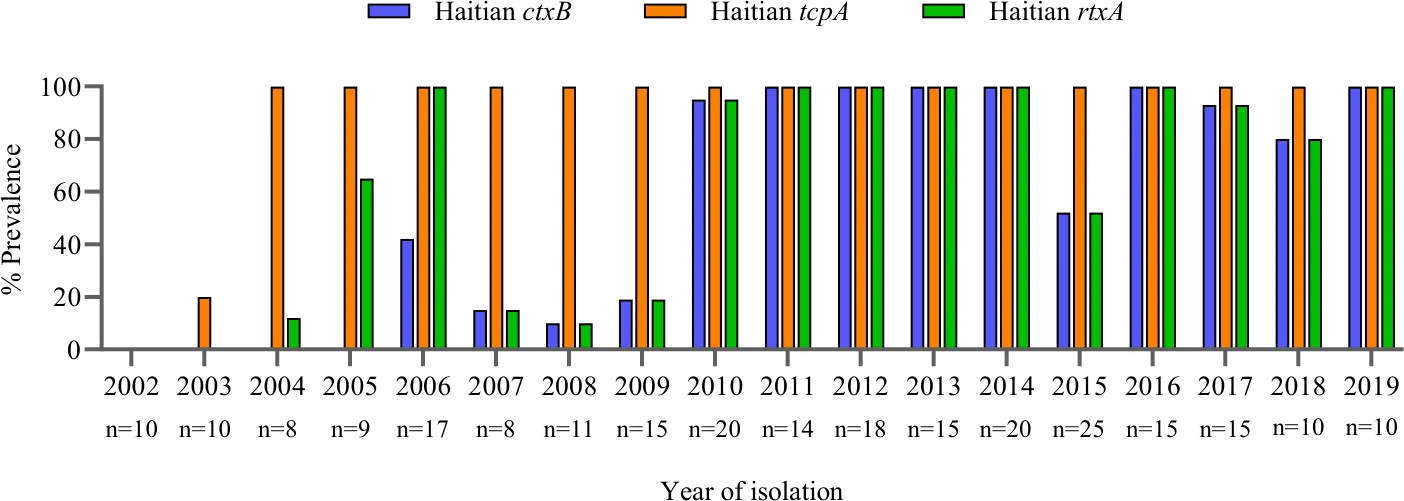
Occurrence of Haitian variant alleles of *ctxB*, *tcpA*, and *rtxA* in *V. cholerae* O1 isolated from 2002 to 2019 in Kolkata. A total of 250 representative strains were tested during this study period. “*n*” denotes the number of strains tested in each year.

These findings underscore the progressive and sustained emergence of the Haitian-type alleles in Kolkata, beginning with the appearance of *tcpA*^CIRS^ in 2003, followed by the spread of *rtxA^o^* from 2004 and *ctxB7* from 2006 (Fig. 2). The temporal distribution of these alleles highlights the dynamic evolution of *V. cholerae* O1 in the region and confirms the efficacy of our multiplex PCR assay in tracking virulence gene profiles across time.

### Multiplex PCR results show full concordance with conventional simplex PCR assays

Results of multiplex PCR were verified with conventional simplex PCR methods using the primers and PCR cycling conditions published previously. All 250 *V. cholerae* O1 clinical isolates were subjected to simplex PCR targeting the *rtxA* using the primer pair rtxA-F/rtxA-R2, which amplifies a 187-bp fragment specific to the Haitian variant *rtxA* (*rtxA^o^*). A total of 175 isolates (70%) tested positive, and these strains also consistently yielded the expected *rtxA^o^* amplicons in the multiplex PCR assay, confirming complete concordance between the two methods. The remaining 75 isolates (30%) that tested negative for the Haitian-specific *rtxA* allele in both simplex and multiplex assays were further examined using El Tor-specific primers (rtxA-F/rtxA-R1) following previously established protocols (18). All 75 isolates tested positive for the El Tor variant of *rtxA*, confirming their genotypic identity.

For the *tcpA*, 232 isolates (92.8%) produced amplicons in both the Haitian-specific multiplex PCR and corresponding simplex PCRs. The remaining 18 isolates (7.2%) were screened using El Tor-specific primers (tcpA-F1/tcpA-El-rev), and all yielded a 156-bp product, confirming the presence of the El Tor allele of *tcpA*. Likewise, 159 isolates (63.6%) yielded the expected Haitian-specific *ctxB* amplicon in the multiplex PCR, which was subsequently confirmed by allele-specific simplex PCR using primers ctxB-F3/Rv-cla. The remaining 91 isolates (36.4%) showed no amplification with Haitian-specific primers in either assay. These isolates were then tested using primers ctxB-F4/Rv-cla targeting the classical *ctxB* allele (17), all of which produced a 191-bp amplicon, confirming the presence of the classical *ctxB* variant. Overall, the multiplex PCR assay demonstrated 100% concordance with traditional simplex PCR methods, affirming its reliability and utility for comprehensive genotyping of circulating *V. cholerae* O1

### Multiplex PCR demonstrates high sensitivity and specificity

To assess the diagnostic accuracy of the multiplex PCR assay, we employed a standard 2 × 2 contingency table by comparing its results with those obtained from conventional simplex PCR assays. The comparative data are summarized in Table 2. Based on this analysis, the sensitivity, specificity, positive predictive value, and negative predictive value of the multiplex PCR were all calculated to be 100%, with a statistically significant *P* value of <0.0001. These results confirm the excellent agreement between the multiplex and simplex PCR assays, demonstrating that the multiplex assay is both highly sensitive and specific for detecting the targeted virulence genes in *V. cholerae* O1 isolates.

**TABLE 2:** Comparative analysis of sensitivity and specificity of newly developed multiplex PCR assay with conventional simplex PCR assay using a two-tailed Fisher’s exact test in GraphPad Prism to calculate the *P* value^*c*^

Multiplex PCR assay	Conventional simplex PCR assay		Total
	Haitian genotypes (at least for one gene^*a*^)	Genotypes other than Haitian (no band in Haitian allele-specific simplex PCR)	
Haitian genotypes (at least for one gene)	232	0	232
Genotypes other than Haiti (no band in Multiplex PCR)	0	18^*b*^	18
Total	232	18	250

^*a*^ At least one gene among the Haitian variant *ctxB*, *tcpA,* and *rtxA.*

^*b*^ These strains were confirmed for the presence of classical genotype of *ctxB* and El Tor genotypes of *tcpA* and *rtxA*, respectively, with the help of simplex allele-specific PCRs.

^*c*^ Sensitivity = 232/(0 + 232) × 100 = 100%. Specificity = 18/(0 + 18) × 100 = 100%. Positive predictive value = 232/(0 + 232) × 100 = 100%. Negative predictive value = 18/(0 + 18) × 100 = 100%. *P* value < 0.0001.

### Comparison with existing methods for the detection and characterization of *V. cholerae*

We have gone through several established methods for the rapid detection and characterization of *V. cholerae*, comparing them with our multiplex PCR assay in terms of speed, sensitivity, specificity, field applicability, cost-effectiveness, and ability to provide genetic information (Table 3).

**TABLE 3:** Comparison of available methods for the detection and characterization of *V. cholerae* isolates

Method	Speed	Sensitivity	Specificity	Genetic information	Field applicability	Detection	Strain characterization	Cost	References
Culture	Slow (24–48 h)	High	High	No	Low	Yes	No	Low	(43)
Rapid diagnostic tests	Fast (15–30 min)	Moderate	Moderate	No	High	Yes	No	High	(44)
Traditional PCR	Medium (3–5 h^*a*^)	High	High	Yes	Medium	Yes	Yes	Medium	(45)
Loop-mediated isothermal amplification	Fast (~1 h)	High	High	Yes	High	Yes	No	High	(46)
Whole-genome sequencing	Slow	Very high	Very high	Yes	Low	Yes	Yes	Very high	(47)
Multiplex PCR	Fast (~2 h^*a*^)	High	High	Yes	–^*b*^	Yes	Yes	Low–medium	This study

^*a*^ Considering DNA has been isolated using commercial kits.

^*b*^ Not determined.

The conventional culture-based approach, while highly specific and sensitive, is time-intensive—requiring approximately 48 h to yield interpretable results (43). In contrast, rapid diagnostic tests, including dipstick assays and loop-mediated isothermal amplification, offer faster results with moderate to high sensitivity and specificity, though they are relatively more expensive than some traditional techniques (44, 46). Standard PCR assays that target virulence genes share similar accuracy levels with our multiplex PCR method. However, they often involve multiple simplex PCR reactions, increasing reagent consumption and necessitating the analysis of several agarose gels. This not only extends the time required but also raises the overall cost (45). Whole-genome sequencing remains the most comprehensive and sensitive approach for strain characterization, providing in-depth genetic information. Nonetheless, WGS is cost-prohibitive for routine use and typically requires several days for data interpretation (47).

In comparison, our multiplex PCR assay offers a balanced and efficient solution. It is highly sensitive and specific, capable of generating genetic insights, and allows for the rapid and cost-effective detection of *V. cholerae*. Notably, it enhances detection accuracy by specifically identifying Haitian genetic markers associated with increased virulence, while also minimizing reagent usage and processing time (Table 3).

## DISCUSSION

*V. cholerae* continuously undergoes periodic genetic rearrangement to better adapt to environmental pressures, resulting in the emergence of increasingly hypervirulent forms (11, 48). Recent cholera epidemics exemplify this adaptive trajectory. Since 1961, the El Tor biotype has been the predominant cause of large-scale outbreaks worldwide. Notably, the catastrophic cholera outbreak in Haiti was driven by hypervirulent strains that carried distinct genetic modifications in key virulence genes, including *tcpA*, *ctxB*, and *rtxA*. These mutations significantly enhanced the pathogenic potential of *V. cholerae* O1.

Following their emergence, *V. cholerae* isolates with Haitian genetic signatures have been reported across several regions, including Nigeria, India, and Mexico (17–19, 35–37). The recent devastating cholera epidemic in Yemen—regarded as the worst in recorded history—was also caused by the isolates possessing the Haitian genetic background (49). Phylogenetic analyses have traced the origins of both the Yemeni and recent Zimbabwean cholera outbreaks to a South Asian lineage (50). In addition to shared genetic traits, several phenotypic traits observed in Yemeni strains had previously been documented in *V. cholerae* isolates from Kolkata, some of which date back over a decade (11, 51).

Given the prominent role of South Asia as a reservoir and transmission hub for genetically diverse and potentially hypervirulent strains, we selected isolates from this region to evaluate the effectiveness of our multiplex PCR assay. Our findings demonstrate that currently circulating *V. cholerae* isolates in Kolkata, which represent a subset of the South Asian population, have carried Haitian-associated genetic markers for decades. These lineages continue to evolve, underscoring the importance of ongoing molecular surveillance. Specifically, we focused on genotypic variations in *ctxB*, *tcpA*, and *rtxA*, essential markers for the early characterization of pathogenic potential.

The multiplex PCR assay developed in this study effectively detected Haitian variant alleles, in concordance with previously published genotypic data, including isolates from 2003 to 2006. Furthermore, we provide novel insights into the virulence gene profiles of *V. cholerae* isolates collected between 2015 and 2019—a period for which such data had not been previously reported. The persistence of Haitian genetic traits in these isolates underscores their ecological stability and potential to drive large-scale outbreaks across diverse geographies.

### Conclusion

Given the persistent global threat posed by cholera, the rapid and precise identification of virulence-associated genes in *V. cholerae* remains a public health imperative. Existing screening methods are often hindered by limitations in speed, cost, specificity, and their ability to effectively characterize virulence potential. The multiplex PCR assay developed in this study addresses these shortcomings by offering a rapid, cost-efficient, and highly informative approach for the detection of pathogenic *V. cholerae* isolates. This assay demonstrates substantial promise for both clinical diagnostics and public health surveillance, facilitating prompt outbreak response and enabling the implementation of targeted control measures.

## Data Availability

The data sets used and/or analyzed during the current study are available from the corresponding author on reasonable request.
